# Characterizing trends in human-wildlife conflicts in the American Midwest using wildlife rehabilitation records

**DOI:** 10.1371/journal.pone.0238805

**Published:** 2020-09-11

**Authors:** Rachel B. Long, Kristi Krumlauf, Anna M. Young

**Affiliations:** 1 Department of Biology & Earth Science, Otterbein University, Westerville, Ohio, United States of America; 2 Ohio Wildlife Center, Columbus, Ohio, United States of America; Universidad Austral de Chile, CHILE

## Abstract

Human-wildlife conflict is difficult to measure, but the analysis of records from wildlife rehabilitation facilities has shown potential as a technique for characterizing human impacts on wildlife. To examine the value of wildlife rehabilitation records for characterizing local human-wildlife conflicts and prevalence of select wildlife diseases, we reviewed 45,668 records representing over 280 species admitted to a wildlife rehabilitation facility over a 10-year period (2005–2014). We identified the most frequently recorded causes of admission for commonly admitted species, and evaluated how causes of admission may vary across taxa throughout the year. Our analyses support the value of wildlife rehabilitation facility data for characterizing some pressures from human-wildlife conflict and select disease trends for certain taxa, as well as utility for informing topics to emphasize in local conservation education efforts. For example, orphaned neonatal wildlife accounted for the largest proportion of admissions to this facility, and highlights a opportunity for conservation education regarding when wildlife is truly orphaned and requires professional intervention. Additionally, domestic dog attack cases accounted for a proportion of admissions comparable to that of domestic cat attacks, demonstrating a need for the conversation surrounding the impact of domestic pets on local wildlife to expand to include dogs in addition to cats.

## Introduction

Interactions between humans and wildlife have become more frequent as a consequence of encroachment, resulting in an increase in the likelihood of human-wildlife conflict events and zoonotic disease transmission [1, 2]. Human-wildlife conflicts—negative interactions between humans and wildlife that pose a real or perceived threat to either party [3]—are substantial causes of wildlife mortality and population decline, and manifest differently across taxa and anthropogenic contexts [4, 5]. Examples of conflicts demonstrated to significantly impact wildlife populations include vehicle strikes [6, 7], mid-flight collisions with windows [8], domestic cat predation [9], and anthropogenic sources of ecological contamination [10, 11].

Quantifying causes of morbidity and mortality in wildlife populations is often difficult, as there are significant logistical challenges associated with *in situ* studies of wildlife health [12, 13]. It can also be challenging to navigate the behavioral biology of wild populations in a minimally invasive manner; high stress events and capture myopathy can be significant welfare risks to surveys of live individuals [14]. Continued innovation of methods that can be used to identify threats to wildlife health with fewer logistical and welfare challenges is necessary [13].

The analysis of records from admissions to wildlife rehabilitation facilities has potential to be a useful technique for characterizing human-wildlife conflicts and disease trends that may be impacting local wildlife, as this method would not be as subject to the challenges of assessing wildlife health in the field as described by Ryser-Degiorgis [13] and Spalding and Forrester [12]. Additionally, there is an impressive number of wildlife rehabilitation facilities located around the world–for example, there are currently over 60 wildlife rehabilitation organizations located in the state of Ohio [15], over 330 wildlife rehabilitation carers in Australia as of 2000 [16], and over 65 wildlife rehabilitation facilities in the United Kingdom [17]. These facilities each frequently admit hundreds to thousands of animals annually—uniquely positioning them to assess pressures faced by their respective local species [16]. The studies to date from wildlife rehabilitation institutions have focused primarily on identifying common causes of morbidity and mortality for specific taxa, including raptors [18–20], reptiles [21], and black cockatoos [22], as well as comparing rehabilitation outcomes [20, 23], causes for admission across taxa, and causes for admission across ecological niches [24]. A broader analysis of wildlife rehabilitation records could be ideal for identifying human-wildlife conflicts of particular significance for conservation, such as the recent study that used these records to demonstrate the impact of domestic cat predation on a variety of native species [25]. Analyses that span longer time periods could also provide insight regarding how causes of morbidity and mortality may fluctuate seasonally—valuable information for epidemiological studies of disease and human-wildlife conflicts [26, 27], as well as conservation education efforts. One such study on Australian wildlife found that admissions peaked in the spring and summer during breeding seasons [28].

The purpose of this study was to examine the value of wildlife rehabilitation facility admissions records for informing understanding of local human-wildlife conflicts and wildlife disease trends by 1) identifying the top reasons for admission to this facility, 2) examining how occurrences as measured by admitted cases may fluctuate monthly and across taxa, and 3) analyzing trends in commonly admitted disease cases. This information could have direct implications for wildlife rehabilitators and conservation educators, as well as offer information relevant to human and domestic animal health [12, 16, 29].

## Materials and methods

This study utilized records from admissions to a wildlife rehabilitation facility, but did not involve direct interaction with or experimental manipulation of live animals. As such, IACUC approval was not required. However, the permission of the wildlife rehabilitation facility to utilize these records for the purposes of this study was sought and granted.

### Study area

We utilized records from admissions to a wildlife rehabilitation facility veterinary hospital in central Ohio, United States of America. This facility was located in a suburban context, with urban and rural areas in near proximity. Animals were regularly admitted from all three contexts, though most frequently from urban and suburban areas. Most admissions (>70%) originated from the county in which this facility was located and the six immediately surrounding counties (Franklin, Licking, Delaware, Union, Madison, Fairfield, Pickaway). Animals were also admitted from more distant regions of Ohio; at least one animal was admitted to this facility from 74 of the 88 counties in Ohio during the span of this study.

### Admissions records databases

We reviewed records spanning a 10-year period (2005–2014), which included 45,668 individuals from over 280 species, including rabies vector species. Each individual admitted to this facility had been assigned a unique record entry, regardless of life stage or being admitted with conspecifics at the same intake event. The records included both animals that were alive on presentation to the hospital as well as animals that were dead on arrival. Admissions records had been recorded in a Microsoft Access database from 2005–2012, and in the Wildlife Incident Log/Database and Online Network (http://www.wild-one.org) database from 2013–2014. The two databases differed in how information was recorded, so consolidation of all records in the time span of this study necessitated standardization to facilitate analysis across the 10-year period.

### Taxonomic group categorization

We assigned each case to one of eight taxonomic groups: mammals, reptiles, amphibians, waterfowl, water/shorebirds, raptors, gallinaceous birds, and songbirds+. We assigned all mammalian, reptilian, and amphibian species to their respective taxonomic categories. We assigned orders Anseriformes, Podicipediformes, and Gaviiformes to the waterfowl taxonomic group, orders Pelecaniformes, Gruiformes, Ciconiiformes (excluding vultures), and Charadriiformes to the taxonomic group of water/shorebirds, the orders Falconiformes, Accipitriformes, Strigiformes, and vulturine Ciconiiformes to raptors, and the order Galliformes to gallinaceous birds. We categorized all passerines and other small bodied terrestrial birds, namely the orders Piciformes, Caprimulgiformes, Apodiformes, and Columbiformes, into the songbirds+ taxonomic group. We classified records where species was not identified as unknown.

### Cause of admission categorization

Information in the records regarding the circumstances that led to an animal being admitted to this wildlife rehabilitation facility was documented according to what was stated by the individuals admitting the animal to the facility and/or derived from physical examinations by a veterinarian upon admission. Causes for admission were numerous and there was variation in how the causes were documented in the records, so to facilitate analysis we created a categorization system that involved assigning each case a broad cause of admission category and a specific subcategory. The broad cause of admission categories we created for the purposes of this study were modelled after the “circumstance” categories utilized by the WILD-ONe database system. We divided some of these categories into multiple categories (ex: we divided WILD-ONe’s “Collision” category into the broad categories of “Collision with Non-moving Object” and “Collision with Moving Object”) to enable us to analyse groups of similar specific causes for admission with more precision. Our list of specific cause of admission categories was formulated based on the more detailed information contained in records related to the circumstances that led to an animal being admitted. These lists of broad cause of admission categories and specific cause of admission subcategories were then applied to all records across both the Microsoft Access and WILD-ONe databases resulting in 83 broad categories and 20 subcategories (S1 Table). If an animal was admitted with multiple injuries, we assigned its cause of admission based on the primary reason the animal was brought to the wildlife hospital. If an animal was admitted with both a disease and an injury–for example, a raccoon (*Procyon lotor*) that had been struck by a vehicle but also exhibited signs of canine distemper virus—we categorized the animal with the cause of admission categories corresponding to both the source of its injury and its disease to ensure that disease occurrences were represented appropriately. This dual categorization was only necessary for 14 cases.

While most records explicitly stated the reasons that individuals were admitted to the hospital and cause for admission categories were assigned accordingly, there were records where the circumstances that resulted in an animal’s injury were either unknown by the individual who admitted the animal, or were not recorded. In unknown cases, it was possible in many instances to reasonably assign a cause for admission based on corroborating information and/or findings from physical examinations–an approach also utilized by other facilities [30]. A frequently encountered example of this involved animals that had been found by the side of a road, but had not been observed or recorded being hit by a vehicle. If a record in this instance contained at least two other items of information (fractures, abrasions, contextual information, etc.) that would implicate a car strike, we assigned the individual the broad category of “Collision with Moving Object” and specific subcategory of “Hit by Vehicle.” If a record stated an individual was “found by road” but there was not sufficient information to implicate a car strike, we assigned the broad category of either “Unknown” if no other relevant information was included, or “Injury Unspecified Cause” and the appropriate subcategory if at least one injury was noted.

For the purposes of this study, causes of admission considered to be human-wildlife conflicts involved animals being admitted due to an adverse direct interaction with a human (ex: vehicle strikes, lawn mowing equipment strikes, or intentionally inflicted trauma) or an adverse indirect interaction with a human or a human-associated phenomenon (ex: window strikes or domestic pet attacks). There are many reasons that wildlife may become orphaned, ranging from misinformed human interferences, the caregiving parent(s) becoming deceased due to a human-wildlife conflict, predation, etc.; the reason neonates become orphaned is rarely known unless the event was directly observed. We have chosen to include animals with the “orphaned” cause of admission designation to be part of the conversation regarding local human-wildlife conflict as it is postulated by wildlife rehabilitators that a noteworthy portion of neonate and juvenile animals admitted as orphans were likely not truly orphaned.

While the prevalence of some diseases can be exacerbated by human influence, such as how close contact with infected individuals at bird feeders or contact with contaminated bird feeders contributes to the spread of mycoplasmal conjunctivis in finches [31], diseases are considered separately from human-wildlife conflicts.

As this wildlife rehabilitation facility is a non-profit organization that relies largely on donations/grants to operate, and as such has limited resources to dedicate to diagnostic testing, some disease cases were identified based on a syndromic approach, where a presumed diagnosis is made based on clinical signs, relevant history, and/or response to treatment. Mycoplasmal conjunctivitis and avian botulism are two examples of diseases in this study where cases were typically identified using this approach, as they have very characteristic clinical signs. Suspected West Nile virus cases were at times presumed based on a syndromic approach, but were typically also supported via serum antibody blood tests as resources allowed. However, it was not indicated in this data set which cases were confirmed and which were not. While this approach is certainly not without limitation and possibility for error, it is common in wildlife rehabilitation facilities across the United States due to similar resource constraints, and does have some merit; syndromic surveillance for WNV was recently described as still potentially conferring value to WNV surveillance efforts [32].

Suspected canine distemper virus cases in mesopredator species such as raccoons were typically confirmed via serum antibody tests. Rabies testing was pursued in mentally inappropriate mesopredators when there was a known situation where another animal or a human may have been exposed.

### Data analysis

To examine how human-wildlife conflict as measured by admissions to this facility may vary seasonally and across taxonomic groups, we assessed changes in mean cases admitted per month via Chi-squared tests. We also described the total number of each taxonomic group admitted, the top 20 species admitted, the top five broad causes for admission, the top five specific causes for admission, and the top three specific causes for admission for each taxonomic group.

To characterize the most common causes for admission to this wildlife rehabilitation facility, we first identified the top four specific causes for admission. For each of the top four specific causes, we assessed the number of cases admitted, the percentage that those cases constituted of all cases admitted to the facility, the taxonomic groups affected, and how mean cases per month fluctuated with Chi-squared tests. For one of the top four specific causes for admission, vehicle strikes, we utilized average daily traffic volume data [33] for the county where this facility was located to evaluate the contribution of monthly and annual changes in traffic volume to trends in vehicle strike cases with linear regression tests.

To examine the potential of wildlife rehabilitation records to contribute to disease monitoring, we assessed the number of cases admitted for five diseases of particular importance to the wildlife rehabilitation facility–canine distemper virus, West Nile virus, avian botulism, mycoplasmal conjunctivitis, and rabies. We identified the species that were affected by each of these five diseases, as well as the percentage of all cases admitted that these diseases constituted. For the most represented of the five diseases, canine distemper virus, we also analyzed fluctuations in mean cases per month and in total cases per year via Chi-squared tests as it was the only disease with a degree of representation in the records that facilitated meaningful analysis (n>100 cases across 10 years).

## Results

### Admissions over time and across taxa

**Fig 1:**
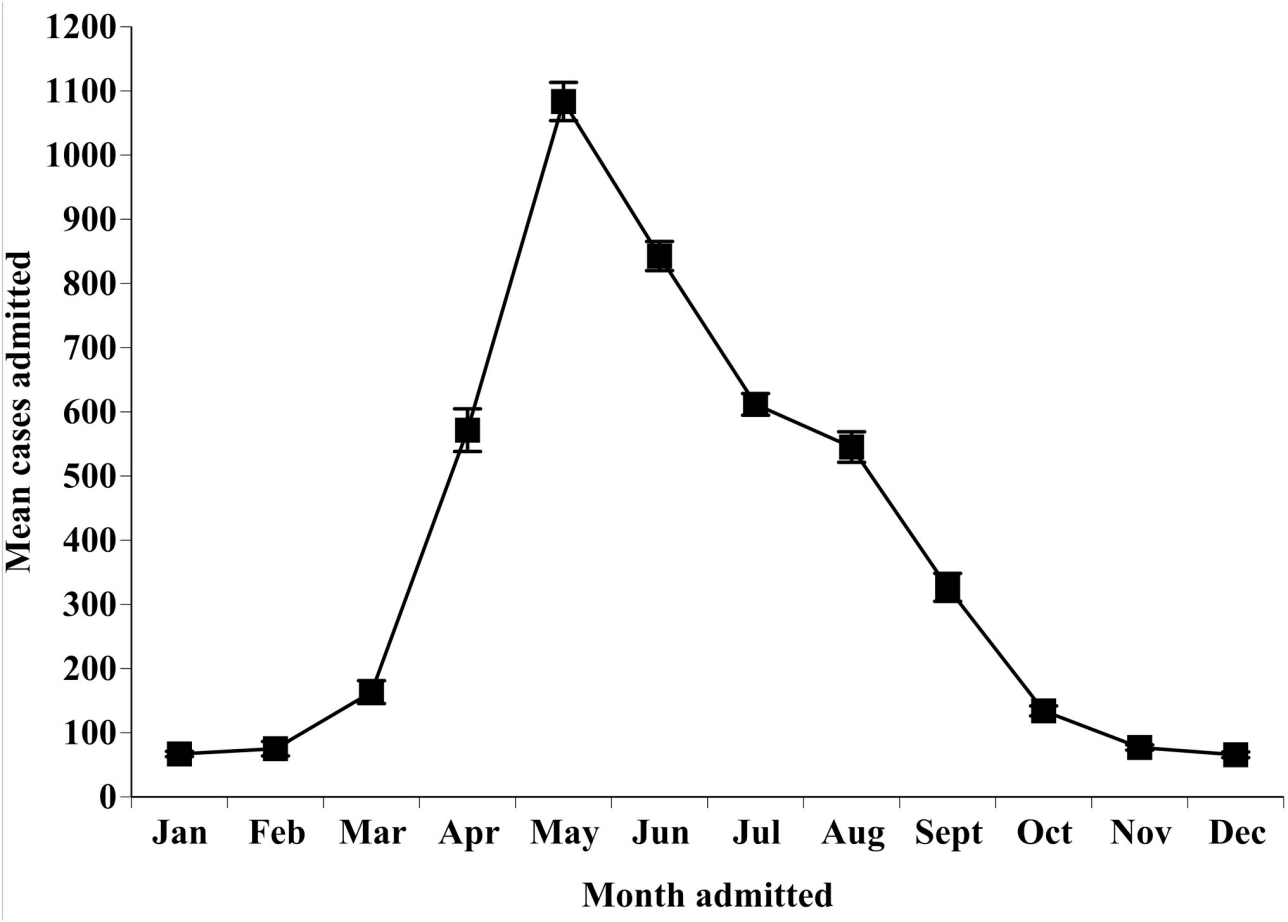
The mean ± SE cases admitted per month to the wildlife rehabilitation facility from 2005–2014.

A total of 45,668 cases were admitted to this wildlife rehabilitation facility from 2005–2014, with a mean of 4,562.7 ± 226.51 cases admitted annually. Of these records, 98% contained sufficient information to be included in some level of analysis and 92% contained sufficient information to be assigned a broad and specific cause of admission category. Total admissions varied across all 10 years ($\chi_{9}^{2}=112.44$, P < 0.001), ranging from 4,105 to 4,848. The mean cases admitted each month fluctuated throughout the year ($\chi_{11}^{2}=3467.74$, P < 0.001). More cases were admitted from May through August than October through March, with May being the peak month for total cases admitted (1,083.5 ± 89.29). The month with the fewest mean cases admitted was December (65.9 ± 13.29); (Fig 1).

Of the eight defined taxonomic categories, mammals were the most frequently admitted, representing over half of all cases (Table 1). Of the 20 species most frequently admitted, seven were mammals, with the eastern cottontail rabbit (*Sylvilagus floridanus*) accounting for nearly one-fourth of all admitted cases. Songbirds+ also accounted for a notable percentage of cases admitted, followed by waterfowl, raptors, and reptiles. Water/shorebirds, amphibians, and gallinaceous birds accounted for only a small portion of all cases admitted, 0.4% collectively.

**Table 1:** The total number and percentage of all admitted cases represented by each taxonomic group, and the total number and percentage of all admitted cases represented by the top 20 most frequently admitted species.

Species	Total Admitted
**Mammals (43 spp.)**	24815 (54.5%)
Eastern cottontail rabbit (*Sylvilagus floridanus*)	10715 (23.5%)
Eastern grey squirrel (*Sciurus carolinensis*)	4831 (10.6%)
Raccoon (*Procyon lotor*)	3192 (6.8%)
Virginia opossum (*Didelphis virginiana*)	3105 (6.8%)
Big brown bat (*Eptesicus fuscus*)	417 (0.91%)
Striped skunk (*Mephitis mephitis)*	407 (0.89%)
Eastern chipmunk (*Tamias striatus*)	373 (0.82%)
**Songbirds+ (135 spp.)**	13111 (28.7%)
American robin (*Turdus migratorius*)	2708 (5.9%)
House sparrow (*Passer domesticus*)	1945 (4.3%)
European starling (*Sturnus vulgaris*)	1443 (3.2%)
Mourning dove (*Zenaida macroura*)	1307 (2.9%)
House finch (*Haemorhous mexicanus*)	626 (1.4%)
Northern cardinal (*Cardinalis cardinalis*)	561 (1.2%)
Common grackle (*Quiscalus quiscula)*	417 (0.91%)
Goldfinch (*Spinus tristis*)	398 (0.87%)
Rock pigeon (*Columba livia*)	386 (0.85%)
**Waterfowl (25 spp.)**	4898 (10.8%)
Mallard (*Anas platyrhynchos*)	3192 (6.9%)
Canada goose (*Branta canadensis*)	1184 (2.9%)
**Raptors (22 spp.)**	1704 (3.7%)
Red-tailed hawk (*Buteo jamaicensis*)	537 (1.2%)
Cooper’s hawk (*Accipiter cooperii*)	397 (0.87%)
**Reptiles (29 spp.)**	641 (1.4%)
**Water/Shorebirds (13 spp.)**	230 (0.51%)
**Amphibians (12 spp.)**	98 (0.22%)
**Gallinaceous (6 spp.)**	56 (0.12%)

The top five most frequently assigned broad causes for admission categories account for 83.5% of all cases admitted, and the top five most frequently assigned specific causes for admission subcategories account for 81.5% of all admitted cases (Table 2). The most assigned broad category and specific subcategory was “Orphaned”, encompassing just over half of all cases admitted. Domestic animal interactions were the second most frequent cause of admission at nearly one in five of all cases admitted; within this broad category, both cat and dog attacks each accounted for close to a tenth of all cases admitted. Collisions with moving objects constituted nearly a tenth of all cases admitted, with vehicle strikes accounting for most of these incidents.

**Table 2:** The five most frequently assigned broad causes for admission, and the two most frequently assigned specific causes for admission corresponding to each.

Causes of Admission	Total Admitted
Orphaned	23201 (50.6%)
Orphaned	23201 (50.6%)
Domestic Animal Interaction	8286 (18.1%)
Cat Attack	4491 (9.8%)
Dog Attack	3755 (8.2%)
Collision with Moving Object	3932 (8.6%)
Hit by Vehicle	3418 (7.5%)
Hit by Lawn Equipment	415 (0.9%)
Injury Unspecified Cause	1519 (3.3%)
Bone Fracture	556 (1.2%)
Wounds/Abrasions	184 (0.4%)
Collision with Non-Moving Object	1180 (2.6%)
Collision with Building/Window	1057 (2.3%)
Collision with Natural Object/Structure	86 (0.2%)
Other Broad Cause of Admission	7613 (16.7%)
Other Specific Cause of Admission	8454 (18.5%)

The most frequently observed specific causes for admission varied across taxonomic groups (Table 3). Being orphaned was the most frequent reason for admission for mammals, songbirds+, waterfowl, water/shorebirds, and gallinaceous birds, and the second most common reason for raptors and reptiles. Vehicle strikes were the top cause of admission for raptors, reptiles, and amphibians. Domestic animal attacks were either the second or third top cause for admission of mammals, songbirds+, reptiles, and gallinaceous birds. Collisions with buildings/windows were the third most recorded cause of admission for songbirds+ and raptors. Amphibians were most frequently admitted due to lawn mower strikes.

**Table 3:** The top three specific causes of admission for each taxonomic group, with total count and percentage of taxon individuals admitted due to each cause, release rate, and mortality.

Taxon	Top Specific Cause of Admission		2^nd^ Specific Cause of Admission		3^rd^ Specific Cause of Admission	
Mammals	Orphaned	13627 (54.8%)	Dog Attack	3272 (13.2%)	Cat Attack	2513 (10.1%)
Songbirds+	Orphaned	6199 (47.4%)	Cat Attack	1805 (13.8%)	Collision with Building/Window	886 (6.8%)
Waterfowl	Orphaned	2968 (60.6%)	Hit by Vehicle	706 (14.4%)	Bone Fracture	95 (1.9%)
Raptors	Hit by Vehicle	436 (25.6%)	Orphaned	179 (10.5%)	Collision with Building/Window	130 (7.6%)
Reptiles	Hit by Vehicle	232 (36.2%)	Orphaned	106 (16.5%)	Dog Attack	29 (4.5%)
Water/Shorebirds	Hit by Vehicle	35 (15.2%)	Orphaned	27 (11.7%)	Bone Fracture	24 (10.4%)
Amphibians	Hit by Lawn Equipment	14 (14.3%)	Bone Fracture	7 (7.1%)	Cat Attack	6 (6.1%)
Gallinaceous	Orphaned	30 (53.6%)	Hit by Vehicle	10 (17.9%)	Dog Attack	4 (7.1%)

### Top causes of admission

We further examined the top four identified specific causes of admission to the wildlife rehabilitation facility: orphaned, dog attacks, cat attacks, and vehicle strikes.

### Orphaned

**Fig 2:**
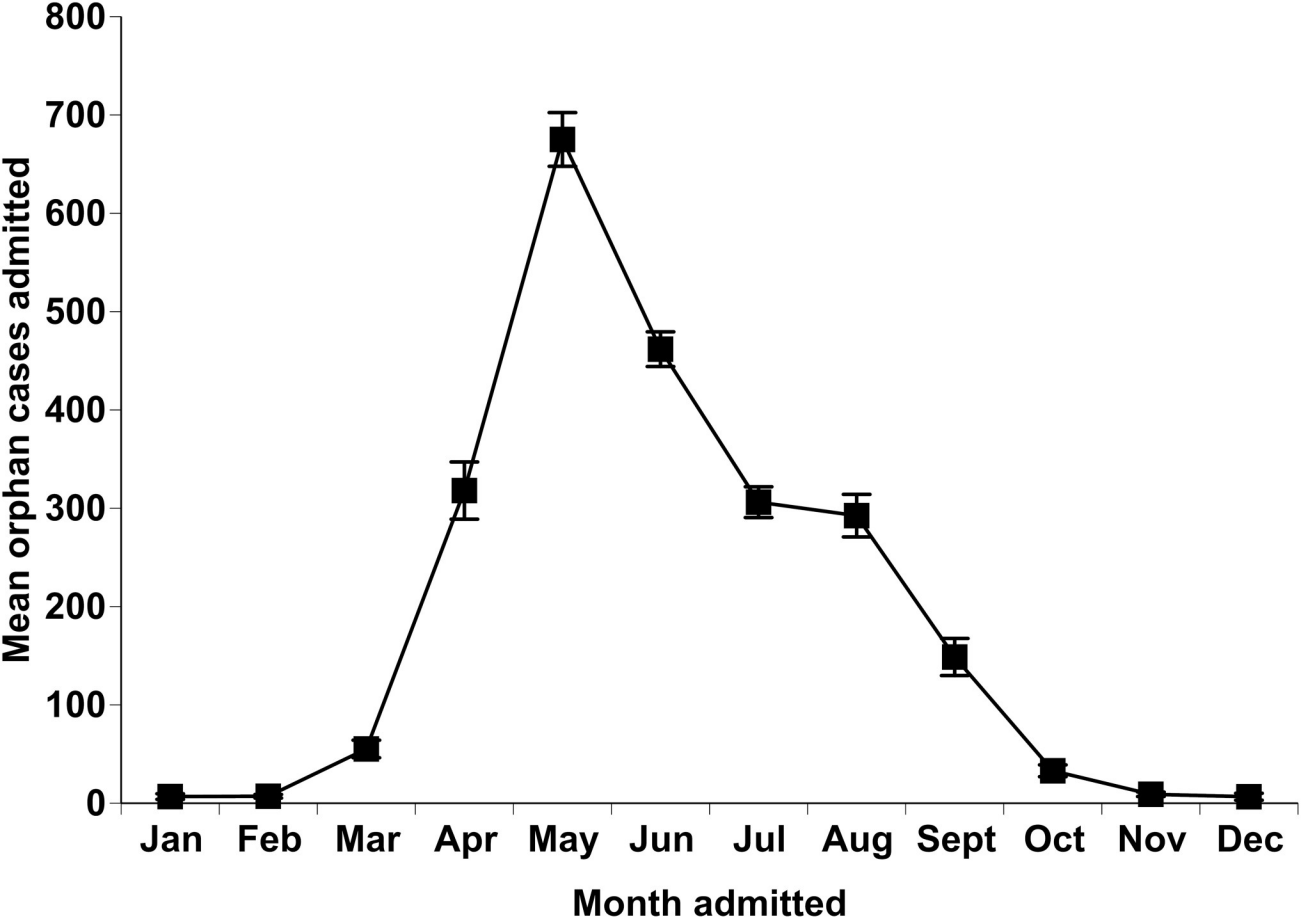
The mean ± SE orphaned cases admitted per month to the wildlife rehabilitation facility from 2005–2014.

A total of 23,201 neonate and juvenile individuals were admitted as having been orphaned, constituting 50.8% of all cases admitted during the study period, a large proportion of admissions during the spring and summer months (Figs 1 and 2), and one of the top three specific causes for admission for seven of the eight taxonomic groups (Table 3). The mean orphan cases varied significantly across months ($\chi_{11}^{2}=2727.59$, P < 0.001), with the peak month being May and the month with the fewest cases being January (Fig 2). The total orphan cases also varied annually ($\chi_{9}^{2}=337.05$, P < 0.001), with the most admitted in 2010 and the fewest admitted in 2007.

### Domestic animal attacks

**Fig 3:**
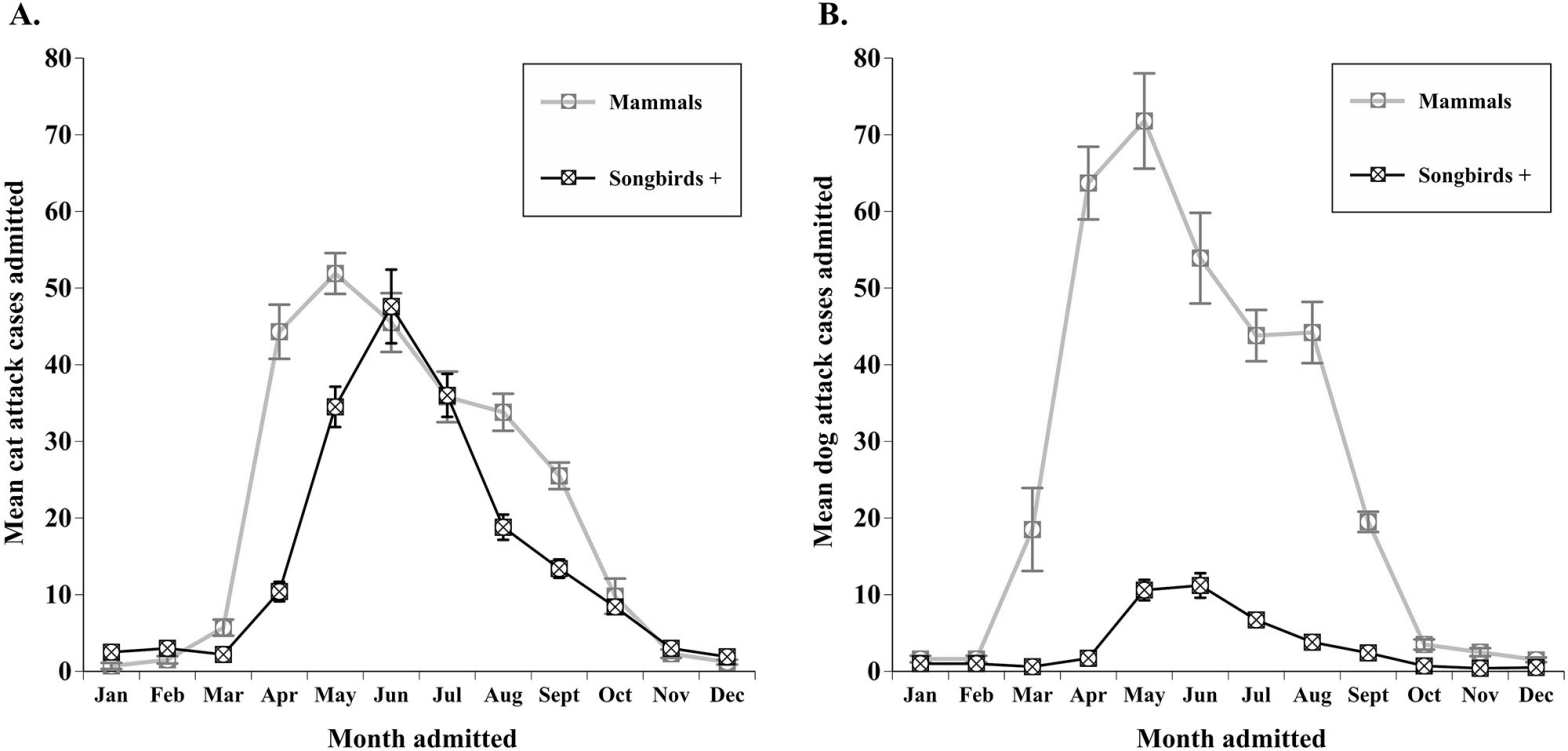
A) The mean ± SE cat attack cases per month, and B) mean ± SE dog attack cases per month admitted to the wildlife rehabilitation facility from 2005–2014 for the top two most impacted taxonomic groups: Mammals and songbirds+.

A total of 4,491 individuals were admitted due to being attacked by a cat, and 3,755 individuals were admitted due to a dog attack. Cat attacks and dog attacks account for comparable percentages of all cases admitted to the wildlife rehabilitation facility (9.8% and 8.2% respectively). The three taxonomic groups most impacted by cat attacks were mammals (2,593 cases), songbirds+ (1,805 cases), and waterfowl (64 cases), collectively representing 69 species. Mammals (3,262 cases), songbirds+ (403 cases), and reptiles (29 cases) were most impacted by dog attacks. Cat attacks impacted 1,413 more songbirds+ individuals than dog attacks, and dog attacks impacted 682 more mammals than cat attacks. The mean cases of cat attacks ($\chi_{11}^{2}=361.99$, P < 0.001) and dog attacks ($\chi_{11}^{2}=323.10$, P < 0.001) varied across months. The peak month for mammal cases admitted due to either cat or dog attacks was May, and the peak month for songbirds+ cases admitted due to either cat or dog attacks was June. The months with the fewest dog attack or cat attack cases admitted were December and January (Fig 3). The total cases of dog attacks ($\chi_{9}^{2}=45.5$, P < 0.001) and cat attacks ($\chi_{9}^{2}=91.63$, P < 0.001) also differed annually, with peak admissions for dog attacks occurring in 2013, peak admissions for cat attacks occurring in 2009, and the fewest cases of either cat or dog attacks being admitted in 2005.

### Vehicle strikes

**Fig 4:**
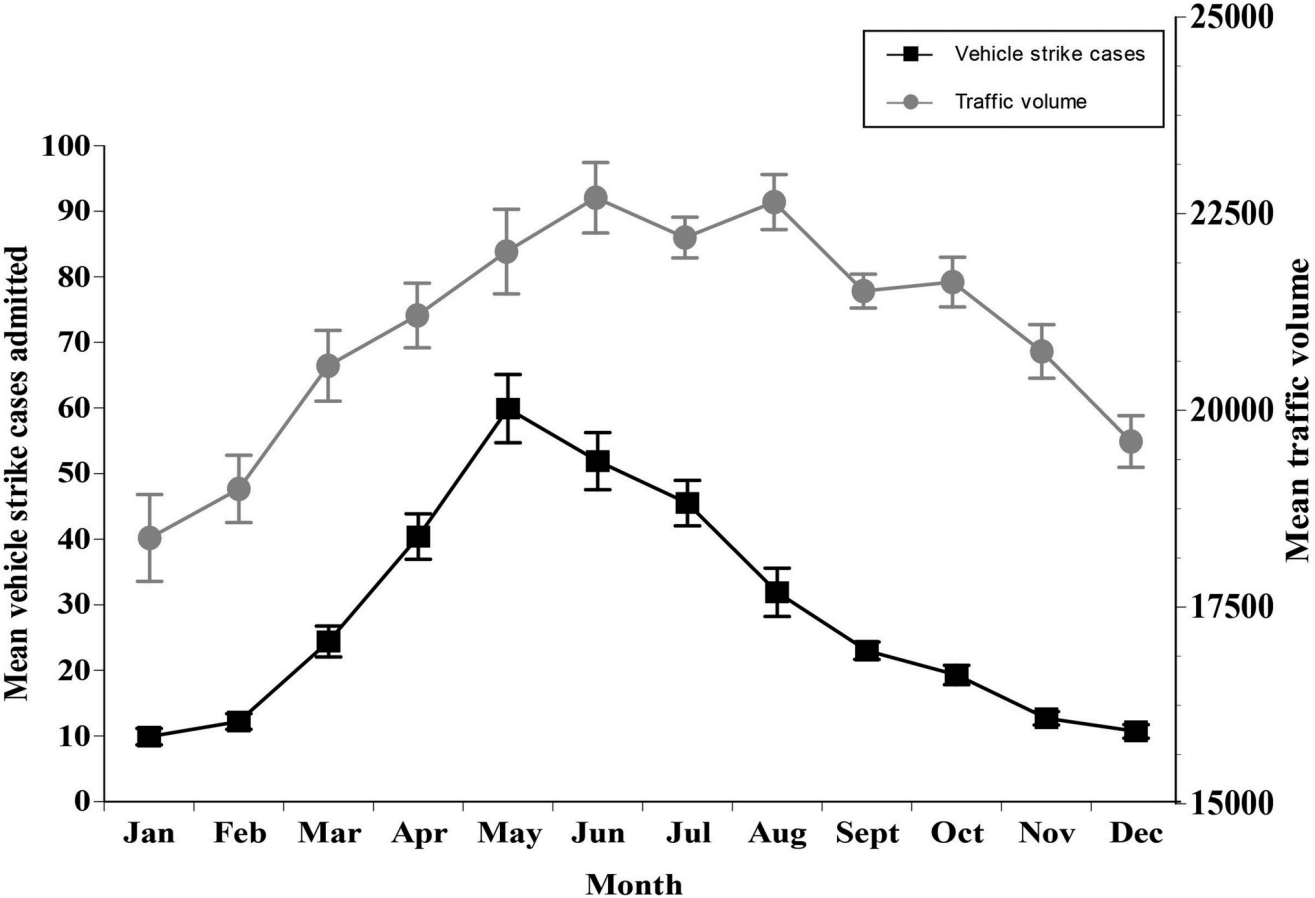
The mean ± SE vehicle strike cases admitted per month to the wildlife rehabilitation facility per year from 2005–2014.

Vehicle strikes represented 8% of all cases admitted to the wildlife hospital. Diverse taxa were admitted due to vehicle strike cases, with Canada geese (*Branta canadensis*), Virginia opossums (*Didelphis virginiana*), eastern cottontail rabbits, mallards (*Anas platyrhynchos*), and eastern gray squirrels (*Sciurus carolinensis*) being the five most admitted species. The mean vehicle strike case admissions varied significantly across months ($\chi_{11}^{2}=115.48$, P < 0.001), and the total cases differed annually ($\chi_{9}^{2}=34.38$, P < 0.001). The peak month for cases admitted due to vehicle strikes occurred in June, and the month with the fewest vehicle strike cases was January (Fig 4). The peak month for mean daily traffic volume in Franklin County, Ohio, USA was June, and the month with the lowest mean daily traffic volume was January (Fig 4). A strong linear relationship was found between mean daily traffic volume per month and mean monthly vehicle strike cases admitted (F_1,10_ = 13.63, P = 0.004, R^2^ = 0.577), but not between mean annual traffic volume and total annual vehicle strike cases admitted (F_1,10_ = 0.416, P = 0.539, R^2^ = 0.056).

### Disease

**Fig 5:**
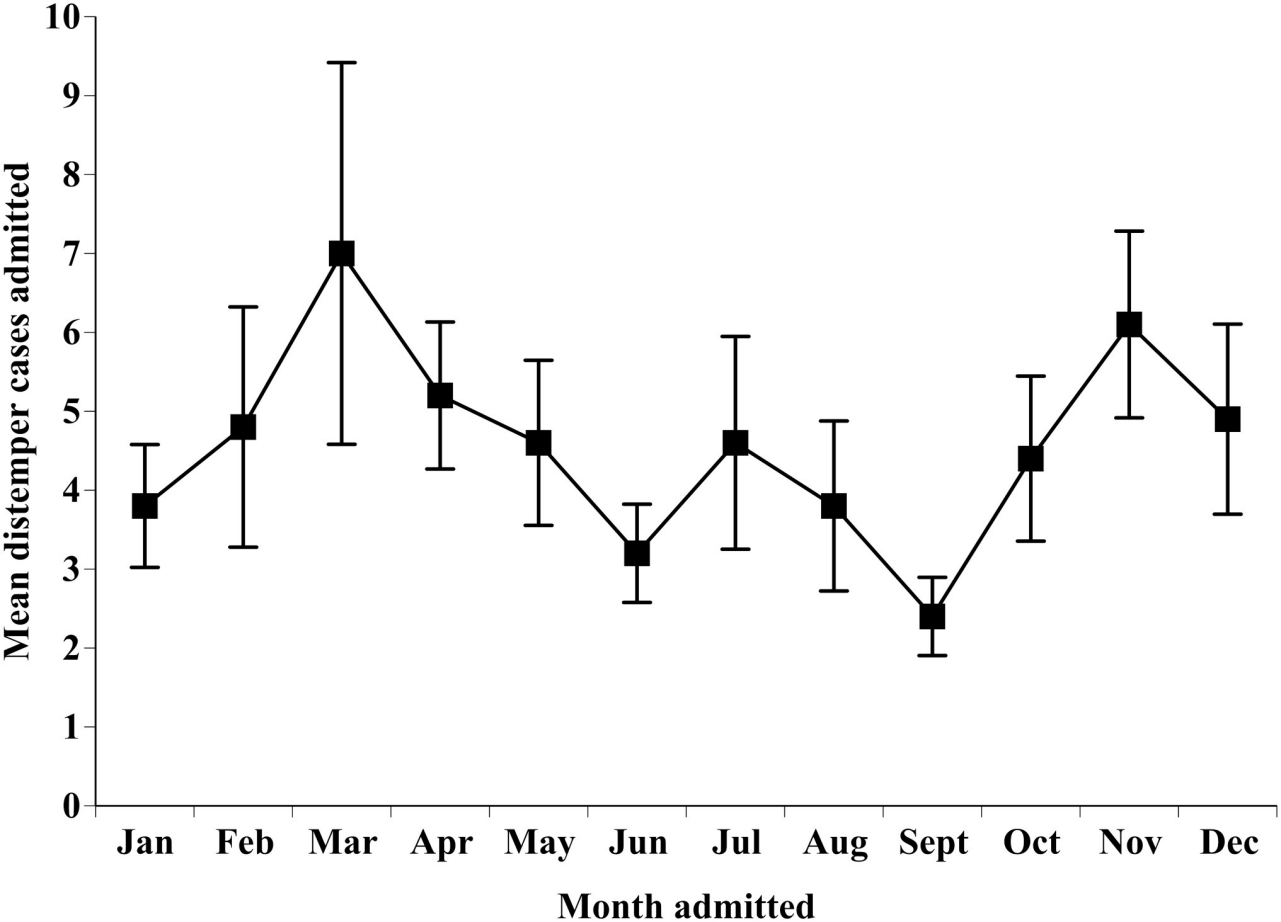
The mean ± SE cases of canine distemper virus admitted per month to the wildlife rehabilitation facility from 2005–2014.

Recorded disease cases accounted for 1,018 (2.23%) of cases admitted from 2005–2014. Canine distemper virus (family Paamyxoviridae, genus *Morvillivirus*) was the most commonly recorded disease state of cases admitted from 2005–2014, with 520 (94%) cases observed in raccoons (*Procyon lotor*) and 30 (5.5%) cases observed in striped skunks (*Mephitis mephitis*). The mean cases of canine distemper per month exhibited some fluctuation (Fig 5), but not to a statistically significant degree ($\chi_{11}^{2}=3.63$, df = 11, P = 0.979). However, across all 10 years there was a fluctuation in total cases admitted annually for both raccoons ($\chi_{9}^{2}=182.77$, df = 9, P < 0.001) and skunks ($\chi_{9}^{2}=34$, df = 9, P = 0.009) with peak years in 2008 for raccoons and 2009 for skunks. Mycoplasmal conjunctivitis (*Mycoplasma* spp.) accounted for 8% of all disease cases admitted, and largely impacted house finches and goldfinches (89% collectively). West Nile virus (family Flaviviridae, genus *Flavivirus*) was recorded for 6% of all disease cases, avian botulism (*Clostridium* spp.) accounted for 4%, and other miscellaneous disease states accounted for 28%. A total of three individuals, all big brown bats (*Eptesicus fuscustested*), tested positive for rabies (family Rhabdoviridae, genus *Lyssavirus*), accounting for 0.3% of disease cases admitted, and 0.007% of all cases admitted to the wildlife rehabilitation facility during the examined time period.

## Discussion

### Overall trends

Our study demonstrates the value of wildlife rehabilitation records for characterizing local human-wildlife conflicts and potentially select disease trends, as well as how occurrences may fluctuate seasonally and impact taxa differently. While our analysis is most directly applicable to the region where this wildlife rehabilitation facility is located, we believe this technique can be applied in other regions as well. A similar study in Australia found that car strikes, dog and cat attacks, and orphans were also top reasons for admissions, although they additionally found entanglement and disease to be highly prevalent [28].

Using the records, we were able to identify what are likely some of the predominant types of human-wildlife conflicts in the central Ohio region, including domestic animal attacks, vehicle strikes, lawn mower strikes, and collisions with buildings, and how they impact taxa differently. It is likely that wildlife rehabilitation facilities have a greater monitoring capacity for some taxa more than others; mammals, songbirds+, and waterfowl constituted the majority of admissions to this facility. It should be noted that distinctive species or subgroups within the taxonomic categories utilized in this study may experience human-wildlife conflict differently; here we focused on broad trends, future studies are needed to tease apart which threats may apply more to specific species or taxonomic subgroupings. Additionally, this facility primarily depends on the public to bring in animals they find, which results in the predominantly admitted species being those that are more frequently seen by people, and those that non-professional individuals are willing to attempt to handle. However, meaningful trends were still able to be discerned for some of the taxa that were admitted, such as mammals being more likely to be admitted due to domestic dog attacks than cat attacks. These findings also suggest utility for future studies that examine how human-wildlife conflicts may vary or be experienced differently by taxonomic groups more specific than those examined in this study.

It was also found that other forms of human-wildlife conflict, such as vehicle strikes, impacted a larger assortment of taxonomic groups. Vehicle strikes were one of the top three specific causes of admission for five of the eight taxonomic groups utilized in this study (Table 3). This suggests that ongoing study and education is needed regarding vehicle strike deterrents such as wildlife-friendly driving practices.

### Top causes of admission

#### Orphaned

Orphaned neonates and juveniles were a primary cause of admission for seven of the eight taxonomic groups admitted, accounting for the largest source of admissions to this facility (Table 2) and highlighting the ongoing need for public education regarding legitimately orphaned wildlife. Orphaned wildlife admissions have constituted a predominant cause for admission in other wildlife rehabilitation facilities as well [19, 34]. This facility has protocols to help screen for neonatal wildlife that may not actually be orphaned, but they mostly entail conversational questioning, and are dependent on the honesty and existing knowledge of the members of the public presenting the animals to the hospital. Thus, increasing the rigor and/or consistency of these screenings may be useful to some degree, but increasing public understanding of topics such as the biology of local neonatal wildlife, how to discern whether found neonates are legitimately orphaned, and local resources that can be contacted for assistance would likely be the more ultimate solution.

#### Domestic animal attacks

Domestic animal attacks demonstrated significant month-to-month fluctuations, likely corresponding with seasonal changes in the biology of local wildlife related to habitat use [35, 36], breeding seasons [37], migration [37, 38], and range size [39, 40]. Domestic animal attack cases may also exhibit this seasonality as during the months of April-August in the northern hemisphere, domestic animals maintained as household pets may be more likely to spend more time outside and thus have a greater likelihood of encountering wildlife. The seasonal influx and thus increased density of migratory songbird species may also be a contributing factor. A similar trend in seasonal rates of domestic cat attacks on wildlife was also reported for another U.S. wildlife rehabilitation facility’s records [25]. This highlights a time of year where it is particularly important to emphasize supervising pets outdoors to decrease the frequency of domestic animal attacks on wildlife.

A similar study that was broader in region, including all North America WILD-ONe data but focusing on cat-wildlife conflicts over a shorter period of time, also determined domestic pet attacks to be a top reason for admission at 14%, comparable to our finding of 18% for this Ohio facility. Unlike our study, they found that birds presented to the facility due to a cat attack more than other taxa, but similarly the species most commonly admitted for cat attacks were the eastern cottontail rabbit, American robin, and eastern gray squirrel [41]. This suggests that our findings are representative of national trends for at least domestic felid attacks.

Our study also suggests that the current conversation regarding the impact of domestic animal attacks on wildlife needs to expand. We found evidence for the previously demonstrated magnitude with which domestic cat predation impacts songbird and mammalian taxa [9, 25, 42], but our findings also illuminate that in terms of total cases admitted, dog attacks exert pressure comparable to cats. It should be noted that cats are primarily predating taxa that are typically considered of higher conservation concern, namely songbirds. Although none of the species in this study were threatened or endangered, many admitted songbird species are protected under the Migratory Bird Treaty Act. However, dogs are impacting small mammals to an extent that is of notable concern from an animal welfare standpoint, and they could pose a conservation threat in regions where endangered small mammals are more prevalent than in central Ohio. While it has been previously suggested that dog attacks may have a noteworthy impact on wildlife populations [43, 44], we provide evidence that dog attacks are another source of anthropogenic pressure on wildlife that should be addressed in wildlife education efforts and accounted for in conservation decision-making for songbird and mammalian taxa.

#### Vehicle strikes

Our findings suggest that changes in traffic volume throughout the year are not the primary driver of the fluctuations observed in vehicle strike cases. While a relationship was found between traffic volume and vehicle strikes across months within a mean year, this was not the case across years. It is likely that seasonal changes in wildlife dispersal, migration, and behavioral biology contribute more significantly to vehicle strikes than the fluctuations in traffic volume recorded in this area. These findings support the relevance of vehicle strikes as a significant source of morbidity and/or mortality in wildlife [45, 46], as well as the need for additional study regarding effective wildlife/vehicle strike prevention measures such as wildlife crossing structures or warning signage for motorists [47]. Additionally, these findings lend support to the value of incorporating mitigation techniques such as wildlife crossing structures into roadway preconstruction planning [47], as well as heightened education efforts during times of the year where changes in wildlife behavioral biology may contribute to increased likelihood of vehicle strikes.

#### Disease

While the mean canine distemper virus cases admitted per month were not found to fluctuate to a statistically significant degree, there was fluctuation in total raccoon and skunk canine distemper cases admitted annually from 2005–2014. These species are known reservoirs of canine distemper virus in this region [48–50]. An increase in admissions of both species due to canine distemper may suggest an increase in overall prevalence of canine distemper cases during those years. This facility also admitted a noteworthy number of cases of Mycoplasma conjunctivitis, an ocular infectious disease primarily affecting finch species that was first observed in Ohio in 1994 [31, 51], throughout the examined time period, These findings, in conjunction with other recent work that has observed annual fluctuations in West Nile virus cases admitted to a Minnesota wildlife rehabilitation facility [32] and characterized European hedgehogs as a natural reservoir for some coronaviruses via surveillance testing of admissions to a facility in northern Italy [52], may implicate value for wildlife rehabilitation admissions for disease monitoring. However, additional research is needed which compares wildlife records and monitored wildlife population disease prevalence before conclusions can be drawn. It should be noted that resources available for diagnostic testing and relatively low rates of diseased individuals being admitted may limit the ability of wildlife rehabilitators to discern small fluctuations in disease occurrences locally [27], and diseases that do not show obvious clinical signs will be underrepresented in this dataset. However, in the event of significant spikes in disease prevalence in local populations, wildlife rehabilitation facilities would be among the first to detect it via abnormal increases in cases admitted, potentially such as those observed in canine distemper cases admitted in 2008 and 2009. This would corroborate speculation in other literature regarding the potential value of wildlife rehabilitation records for wildlife disease monitoring [19, 27, 53].

While rabies is one of the diseases of greatest public health concern, it was only confirmed in three cases out of over 45,000 admitted across 10 years. This could be due to the public being less likely to approach animals exhibiting signs that can be associated with rabies, but is also likely a reflection of the low prevalence observed in this region [54].

### Implications for human-wildlife conflict monitoring and conservation education

While wildlife management challenges are multifaceted, wildlife rehabilitation facilities could feasibly serve as one piece of the puzzle by contributing information regarding human-wildlife conflict and some disease trends in urban and suburban areas broadly, or more specifically by taxon. A standardized system of record keeping would optimize the potential effectiveness of rehabilitators as a resource in this way. Progress has been made with the creation of standardized national databases such as WILD-One (http://www.wild-one.org/), Wildlife Rehabilitation MD (http://wrmd.org/), and state-specific online databases, but there is a need for cohesiveness between organizations and public health agencies regarding data collection [53]. Some of the most significant challenges for wildlife health surveillance are related to the lack of confirmed cases, underreporting of confirmed cases, and lack of infrastructure to facilitate assembling records for surveillance [55]. An increase in federal financial support of wildlife rehabilitation facilities could enable increased staff power, resources for disease-related diagnostics, and a cohesive nationwide database. This infrastructure would facilitate more uniform, cohesive monitoring of human-wildlife conflicts and disease occurrences nationally and internationally, and potentially enable wildlife rehabilitation facilities to contribute to monitoring of these trends on both local and global scales. Additionally, our findings highlighted several needs for public education outreach efforts–namely, the impacts of both domestic cat and dog predation on wildlife, how to recognize when neonatal wildlife is truly orphaned and resources to contact for professional intervention, time periods throughout the year when different human-wildlife conflicts are most likely to occur, and affected wildlife taxa. The identification of such trends and subsequent development of relevant public outreach campaigns has been shown to contribute to wildlife population recovery and maintenance [43], and our findings suggest that wildlife rehabilitation facilities are uniquely positioned to contribute to these efforts.

## Supporting information

S1 Table All broad and specific causes for admission. A complete list of the broad specific causes for admission and the corresponding specific causes for admission that were used to categorize the admissions records from a wildlife rehabilitation facility in the Midwest, USA.
(DOCX)
