# Supplementary material for: Characterizing trends in human-wildlife conflicts in the American Midwest using wildlife rehabilitation records
Source: PLoS One. 2020 Sep 11;15(9):e0238805. doi: 10.1371/journal.pone.0238805 (PMC7485781; doi:10.1371/journal.pone.0238805)
Supplement: S1 Table — A complete list of the broad specific causes for admission and the corresponding specific causes for admission that were used to categorize the admissions records from a wildlife rehabilitation facility in the Midwest, USA. (DOCX) [file pone.0238805.s001.docx]

| Broad Cause of Admission | Specific Cause of Admission |
| --- | --- |
| Burn | Set on Fire  Other Burn |
| Collision with Moving Object | Hit by Vehicle  Hit by Train  Hit by Lawn Equipment  Hit by Airplane  Hit by Other Moving Object |
| Collision with Non-moving Object | Collision with Building/Window  Collision with Natural Object/Structure  Collision with Power Lines  Collision with Wind Turbines  Collision with Other Non-moving Object |
| Collision with Unspecified Object | Collision with Unspecified Object |
| Disease | West Nile Virus  Canine Distemper Virus  Avian Poxvirus  Rabies  Avian Botulism  Mycoplasma Conjunctivitis  White Nose Syndrome (confirmed)  Infected Lacerations/Wound(s)  Respiratory Infection (upper and/or lower)  Other Neurologic Disease  Other Non-neurologic Disease  Unidentified/Unspecified Disease |
| Domestic Animal Interaction | Cat Attack  Dog Attack |
| Electrocution | Electrocution |
| Human Attack Interaction | Non-projectile Attack by Human |
| Inappropriate Human Possession | Kept as Pet  Taken with Intent to Rescue/Raise |
| Injury with Unspecified Cause | Bone Fracture  Head Trauma  Crop Injury  Neck/Spinal Injury  Facial Injury  Ocular Injury  Wounds/Abrasions  Unspecified Injury  Congenital Defect |
| Non-domestic Animal Interaction | Same Species Wildlife Attack  Different Species Wildlife Attack |
| Non-trap Entrapment/Entanglement | Entangled in Fishing Line  Entangled in Other String  Trapped in Sports or Landscaping Net  Trapped in Other Net  Trapped in Litter/Garbage  Trapped in Pool  Trapped in Building  Trapped in Garbage Receptacle  Trapped in Window Well  Trapped in Vehicle  Oil/Grease Contamination  Trapped in Fence  Trapped in Gutter  Trapped in Mailbox  Other Non-trap Entrapment |
| Orphaned | Orphaned |
| Projectile Injury | Gunshot  BB Gun  Paintball Gun  Bow/Arrow  Non-weapon Projectile |
| Toxicity | Lead Toxicity  Landscaping Chemicals Toxicity  Rodenticide Toxicity  Pesticide Toxicity  Unspecified Toxicity  Ornamental Plant Toxicity  Pool Chemical Toxicity |
| Trap Entrapment/Entanglement | Trapped in Humane Cage Trap  Trapped in Glue Trap  Trapped in Grease Trap  Trapped in Leghold/Snare Trap  Trapped in Mouse Trap  Trapped in Mole Trap  Trapped in Other Trap |
| Unspecified/Unknown | Unknown |
| Unspecified Attack Victim | Attack by Unspecified source |
| Unspecified Entrapment | Unspecified Entrapment |
| Weather Event | Precipitation Weather Event  Extreme Temperature Weather Event  Wind Weather Event  Other Weather Event |
